# Patient Clinical Outcomes in Standalone Versus a Combined Ophthalmology-rheumatology Uveitis Clinic

**DOI:** 10.1186/s12348-022-00314-1

**Published:** 2022-11-07

**Authors:** Bing X. Ross, Samantha Habhab, Sarah Syeda, Ahmad Baiyasi, Ilyes Benchaala, Chinwenwa Okeagu, Joshua Barbosa, Jacob Im, Kim Le, Xihui Lin

**Affiliations:** 1 Kresge Eye Institute, Department of Ophthalmology, Wayne State University School of Medicine, Detroit, MI USA; 2 Department of Rheumatology, Wayne State University School of Medicine, Detroit, MI USA; 3 Department of Ophthalmology, Henry Ford Hospital, Detroit, MI USA; 4 Department of Ophthalmology, Ascension Macomb-Oakland Hospital, Warren, MI USA

**Keywords:** Uveitis, Combined clinic, Rheumatology, Steroid sparing therapy, Immunomodulatory therapy

## Abstract

**Background:**

To evaluate uveitis care outcomes in standalone versus a combined ophthalmology-rheumatology clinic.

**Methods:**

Participants were patients aged 18 years and older with a minimum 12-month history of chronic uveitis prior to being referred to the combined uveitis clinic at Kresge Eye Institute and who were treated in the combined clinic for at least 6 months. Best corrected visual acuity (BCVA), objective markers of inflammation, and achieving targeted dose of immunomodulatory therapy (IMT) were compared in the cohort of uveitis patients 6 months prior to and after the initial evaluation in the combined clinic.

**Results:**

Sixty-six percent of study participants were female with a mean age of 51.5 years. BCVA improved from 0.58 logMAR (Snellen: ~20/74) at the initial combined clinic visit to 0.50 logMAR (Snellen: ~20/63) 6 months after the first combined visit (*p* = 0.0137). The establishment of the combined uveitis clinic led to higher frequency of patients at target dose of IMT: an increase from 49.0% at 6 months prior to the combined visit to 70.1.4% and 79.8% at the initial combined visit and 6 months after the combined visit, respectively.

**Conclusion:**

A combined model of management for chronic uveitis patients wherein rheumatological services are coupled with ophthalmic care leads to improvement in patient clinical outcomes and achieving target therapy.

## Introduction

Uveitis is a rare ocular condition characterized by intraocular inflammation, the etiology of which can be autoimmune or secondary to infections [1]. The prevalence of uveitis is estimated to approach 133 per 100 000 [2–5]. Repeated active inflammation can irreversibly damage ocular structures and eventually cause significant deterioration in vision [6]. In addition, the majority of patients who suffer from uveitis are in their working age and it can cause significant decrease in productivity [7]. Uveitis is reported to be the fifth or sixth leading cause of blindness in working age populations in developed countries [8–10]. Therefore, prompt and proper management of uveitis is critical to minimize vision loss and medical burden in patients.

The goal of noninfectious uveitis treatment is to suppress ocular inflammation to prevent further damage to the ocular visual system [7]. Corticosteroids are the mainstay of treatment for uveitis since their initial use in the 1950s [11]. However, due to the local and systemic side effects of prolonged corticosteroid usage, treatment of noninfectious uveitis frequently requires systemic steroid sparing immunomodulatory therapy (IMT). The Multicenter Uveitis Steroid Treatment (MUST) Trial 7-year follow-up study demonstrates that the usage of systemic IMT results in improved outcomes compared to local steroidal therapy [12]. The Fundamentals of Care for UveitiS (FOCUS) global initiative also recommends a similar approach to treat noninfectious uveitis [13].

The management of steroid sparing systemic IMT is complex and frequently beyond the scope of practice for many ophthalmologists. Furthermore, uveitis is frequently associated with underlying systemic diseases that require further workup and treatment [13, 14]. Therefore, many uveitis patients are co-managed with rheumatologists. As a consequence, these patients require frequent visits to both ophthalmologists and rheumatologists. For the working-age population, this represents a significant burden especially if a patient does not have adequate social support. In our inner-city population in Detroit, patients are often unable to attend all their appointments, which can be a barrier to achieving the target dose of IMT.

In response, we implemented a multidisciplinary model of non-infectious uveitis management and established a combined ophthalmology-rheumatology uveitis clinic at the Kresge Eye Institute where patients were simultaneously managed by a rheumatologist and a uveitis specialist. The goal of this study was to evaluate patient clinical outcomes of the combined ophthalmology-rheumatology uveitis clinic and to compare them to those before the establishment of the clinic. We found the combined clinic led to more patients at target dose of IMT and a potential improvement of ocular inflammation and a reduction of systemic steroid usage.

## Materials and methods

### Study design

This study was a retrospective analysis of patients co-managed by an ophthalmologist from the Kresge Eye Institute and a rheumatologist from the Department of Rheumatology of Wayne State University in the setting of a combined uveitis clinic. This project was approved by the Institutional Review Board of Wayne State University and performed according to the tenets of the Declaration of Helsinki. Patient written consent was not required.

### Inclusion and exclusion criteria

Patients aged 18 years and older with a minimum 12-month history of chronic uveitis prior to being referred to the combined uveitis clinic at the Kresge Eye Institute and who were treated in the combined clinic for at least 6 months from July 2018 through June 2019 were included. Patients were excluded if there was no examination data 6 months prior to implementing the combined uveitis clinic or 6 months after their initial visit to the combined clinic, or if they were not evaluated by the rheumatologist.

### Kresge uveitis clinic logistics

Only patients with immune-mediated uveitis or ocular inflammatory diseases and on corticosteroid and/or IMT were referred to the combined uveitis clinic. Prior to the establishment of the ophthalmology-rheumatology combined uveitis clinic, patients were seen by an ophthalmologist trained in uveitis and rheumatologists separately at their respective clinics. In the combined clinic, patients were first seen by the ophthalmologist and then the rheumatologist in a dedicated “uveitis suite” of the Kresge Eye Institute. When IMT was recommended, the choice and target dosage of the IMT was determined by the ophthalmologist. The rheumatologist managed the prescription and side effects of the IMT as well as the systemic rheumatic diseases. In situations in which the rheumatologist recommended a different treatment plan, the two physicians would consult and come to a consensus.

### Data collection

Baseline recorded data included patient demographics such as age, gender, and race. Biomicroscopic examination of the eyes was carefully performed in each patient by the ophthalmologist. The following variables were registered in the electronic medical record system: best-corrected visual acuity (BCVA) converted from Snellen to logMAR scale, location of uveitis (anterior, intermediate, posterior, panuveitis, or scleritis), and diagnosis (association with systemic autoimmune diseases, or idiopathic). The diagnoses of systemic diseases were established by primary care physicians or rheumatologists. Diagnosis of sarcoidosis required tissue biopsy showing non-caseating granulomatous inflammation. The anterior chamber (AC) cells and flare were graded according to the Standardization of Uveitis Nomenclature (SUN) Working Group Grading Scheme for Anterior Chamber Cells and Flare, respectively [15]. The vitreous haze was graded by the Nussenblatt Scoring System [16]. Optical coherence tomography (Cirrus 5000; Carl Zeiss Meditec, Dublin, CA) was performed to evaluate the thickness of the central retina area. Patients with central retinal thickness greater than 315 μm with intraretinal and/or subretinal fluid shown in OCT were considered to have macular edema [17]. Treatment plans recorded whether patients were on corticosteroids and/or IMT, routes of pharmacologic administration, types of pharmacotherapies, and dosage. Target dose of corticosteroids was defined as ≤ 7.5 mg/day, and target doses of IMT were defined as > 15 mg/week for oral Methotrexate, > 2 g daily for Mycophenolate mofetil, > 2 mg/kg/day for Azathioprine [18]. Biological agents were considered at target dose based on clinician assessment.

The primary endpoint was the percentage of patients at target dose of IMT. Other endpoints included daily oral steroid dose, BCVA, and objective markers of inflammation, including AC cells and flare, vitreous haze, and central retinal thickness.

### Statistical analysis

Samples were described by using mean and standard error for continuous variables. Continuous data were analyzed using paired Student’s T test, and categorical data using Fisher’s Exact Test with Prism 9 software (GraphPad Software, Inc., San Diego, CA). *P* value < 0.05 was considered statistically significant.

## Results

### Patient demographics

A total of 135 eyes of 74 patients were included in this study. The mean age of the patients at the initial combined visit was 51.5 ± 12.8 years (range: 20.2–92.4 years) with 66.2% female. The predominant race of patients was African American (85.1%). Panuveitis was the most frequent location of uveitis in our study population (33 patients, 44.6%), followed by anterior and posterior uveitis. Idiopathic uveitis accounted for 59.5% of patients. Uveitis associated with systemic diseases was diagnosed in 40.5% of patients. The demographic and uveitis breakdown are summarized in Table 1.

**Table 1:** Patient demographics and characteristics

Category	Value
Patients (n)	74
Females (n, %)	49 (66.2)
Age (mean±SD)	51.5±12.8
Race (n, %)	
African American	63 (85.1)
Caucasian	7 (9.5)
Asian	1 (1.4)
Middle Eastern	1 (1.4)
Other/not specified	2 (2.7)
Uveitis location (n, %)	
Anterior	20 (27.0)
Intermediate	3 (4.1)
Posterior	11 (14.9)
Panuveitis	33 (44.6)
Scleritis	7 (9.5)
Uveitis association (n, %)	
Idiopathic	44 (59.5)
Sarcoidosis	13 (17.6)
HLA-B27	5 (6.8)
Multiple Sclerosis	3 (4.1)
Vogt-Koyanagi-Harada	3 (4.1)
Systemic Lupus Erythematosus	2 (2.7)
Juvenile Idiopathic Arthritis	2 (2.7)
Behçet’s disease	1 (1.4)
HLA-A29	1 (1.4)

### Inflammation status of the eyes

**Fig. 1:**
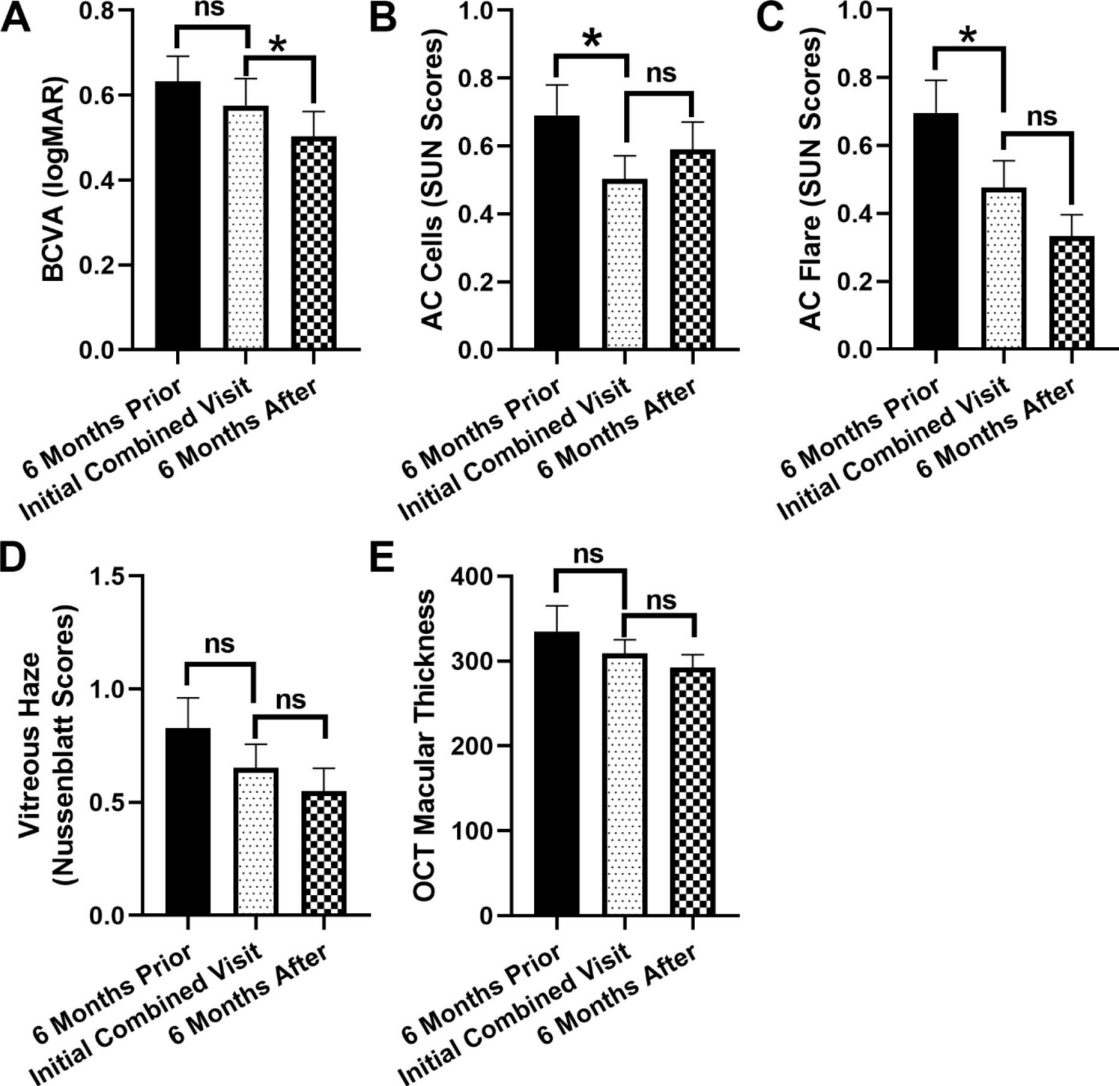
Ophthalmic exam variables evaluated at the initial combined uveitis clinic and 6 months prior to and after the initial combined visits. **(A)** Best corrected visual acuity (BCVA) in log MAR scale. **(B&C)** Anterior chamber (AC) cells and flare graded based on the Standardization of Uveitis Nomenclature (SUN) Working Group Grading Scheme for Anterior Chamber Cells and Flare. **(D**) Vitreous haze graded by the Nussenblatt Scoring System. When evaluating vitreous haze, patients with anterior uveitis and scleritis were excluded from the analysis (see Results for more detail). **(E)** Optical coherence tomography (OCT) was performed to evaluate the thickness of the central macula. Paired Student’s T test. * *p* <0.05, ns: not significant

Figure 1 shows the quantifiable objective data from 6 months prior to being referred to the combined clinic, the first combined clinic visit, and six months after being treated in the combined clinic. There was a statistically significant improvement of BCVA from 0.58 logMAR (Snellen: ~20/74) to 0.50 logMAR (Snellen: ~20/63) after 6 months of the combined clinic (*p* = 0.0137) (Fig. 1 A). AC cell grade, which reflects active inflammation in the eyes, was significantly reduced at the initial combined visit compared to the 6 months prior visit (SUN scores: 0.50 vs. 0.69, *p* = 0.0406) (Fig. 1B). Significant improvement of AC flare grade was also observed between the 6 months prior visit and the initial combined visit (0.70 vs. 0.48, *p* = 0.0272) (Fig. 1 C). No significant difference was detected in AC cell or flare grade between the initial combined visit and the 6 months post visit. When evaluating vitreous haze, patients diagnosed with anterior uveitis and scleritis were excluded from the analysis because their pupils were either not dilated during the visits or the posterior segments could not be viewed due to pupillary synechiae. A decrease in vitreous haze was detected between the three visits; however, the decrease was not statistically significant (Fig. 1D). Macular edema is one of the structural complications of uveitis and can be evaluated by OCT measuring macular thickness in micrometers [15]. We found the means of macular thickness were progressively smaller between the three visits (334.8 vs. 309.1 vs. 292.4 μm). However, the changes were not statistically significant (Fig. 1E).

### Systemic immunosuppressive therapies

Table 2 shows the types of IMT at these three time points. The most frequently prescribed immunomodulatory agents in either ophthalmologist only clinic or the combined clinic were methotrexate, adalimumab, and mycophenolate mofetil. At the initial combined visit, more patients were on IMT compared to the 6 months prior visit, 55.4% vs. 31.1%, respectively. In addition, the initial combined visit promoted the usage of additional immunomodulatory agents: 4 more medications (hydroxychloroquine, dimethyl fumarate, leflunomide, and secukinumab) were introduced in patient treatment plans. Adjustment of dosages and types of immunomodulatory agents was observed at the 6 months post visit. These data demonstrate that the combined effort led to increased utilization and variety of IMT.

**Fig. 2:**
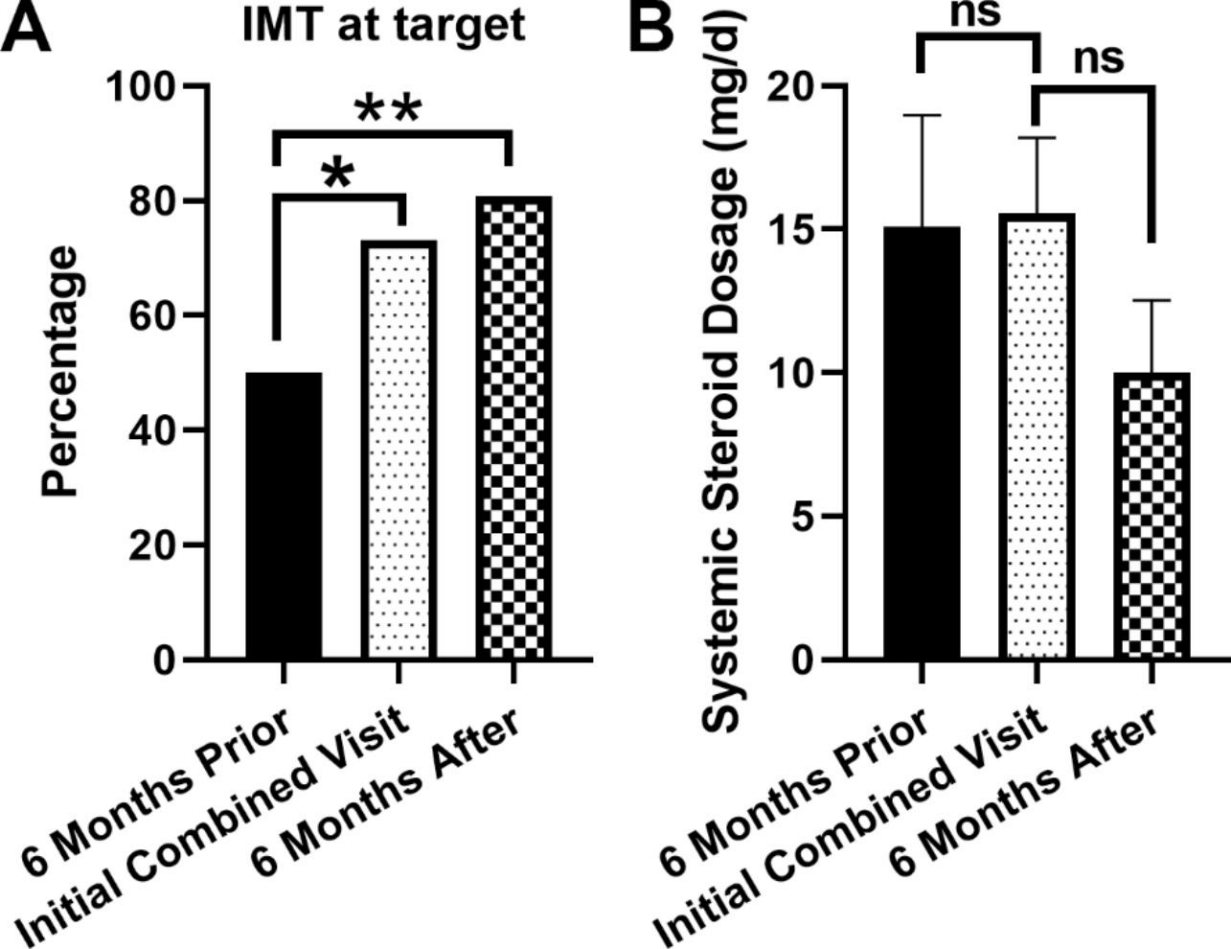
Comparison of the frequency and dosage of systemic immunosuppressive therapies used in patients between the initial combined visit and 6 months prior to and after the initial combined visits. **(A)** The frequency of patients at target dosage of systemic immunomodulatory therapy (IMT). Fisher’s exact test. * *p* <0.05. ** *p* <0.01. **(B)** The average of daily systemic corticosteroid dosage at the three visits. Paired Student’s T test. ns: not significant

The percentage of patients at target dose of IMT and the average daily oral steroid dosages are shown in Fig. 2. Compared to 6 months prior to being treated in the combined clinic, the percentage of patients reaching target dose of the IMT increased from 49.0 to 70.1.4% and 79.8% at the initial combined visit and 6 months after the combined visit, respectively. The average systemic prednisone dose also decreased from 15.1 mg to 10 mg from 6 months prior to 6 months after the combined visit.

**Table 2:** The number and frequency of patients on individual immunomodulatory agent

Immunomodulatory therapy (IMT)	N (%)
**6 months prior**	
Not on IMT	51 (68.9)
Methotrexate	17 (23.0)
Adalimumab	6 (8.1)
Mycophenolate mofetil	5 (6.8)
Azathioprine	3 (4.1)
Infliximab	3 (4.1)
Tacrolimus	2 (2.7)
Cyclosporine	1 (1.4)
Sulfasalazine	1 (1.4)
Ocrelizumab	1 (1.4)
Etanercept	1 (1.4)
**Initial combined visit**	
Not on IMT	33 (44.6)
Methotrexate	26 (35.1)
Adalimumab	11 (14.9)
Mycophenolate mofetil	10 (13.5)
Infliximab	5 (6.8)
Azathioprine	4 (5.4)
Tacrolimus	1 (1.4)
Cyclosporine	1 (1.4)
Sulfasalazine	1 (1.4)
Hydroxychloroquine	1 (1.4)
Dimethyl fumarate	1 (1.4)
Leflunomide	1 (1.4)
Secukinumab	1 (1.4)
Ocrelizumab	1 (1.4)
Etanercept	1 (1.4)
**6 months after**	
Not on IMT	34 (45.9)
Methotrexate	23 (31.1)
Adalimumab	11 (14.9)
Mycophenolate mofetil	9 (12.2)
Azathioprine	6 (8.1)
Infliximab	4 (5.4)
Leflunomide	2 (2.7)
Sulfasalazine	1 (1.4)
Dimethyl fumarate	1 (1.4)
Tocilizumab	1 (1.4)
Golimumab	1 (1.4)

## Discussion

Uveitis is commonly associated with systemic rheumatologic conditions which require assessment and management by rheumatologists [13, 14]. In addition, the use of IMT to manage uveitis and/or the associated systemic diseases requires the expertise of rheumatologists. This often necessitates multiple separate office visits with the potential of lost communication and poor patient follow-up. A combined uveitis clinic potentially lessens the patient burden of visits to multiple providers, minimizes potential delays in therapy, and reduces poor clinical outcomes. In this study, we evaluated patient clinical outcomes of a combined uveitis clinic in which an ophthalmologist and a rheumatologists managed patients simultaneously, and to compare them to those before the establishment of the clinic. We found the combined effort led to increased utilization of IMT and more patients at target dose of IMT, with a potential improvement of ocular inflammation and reduction of systemic steroid usage.

Logistically, it is difficult to establish a combined ophthalmology-rheumatology clinic, but when established, the treatment outcomes seem favorable. The Horst-Bruinsma group in the Netherlands utilized a similar approach with multidisciplinary team meetings and a specialized ocular rheumatology outpatient clinic, to which ophthalmologists could refer patients [19]. They reported that these forms of multidisciplinary team collaboration generates favorable clinical outcomes: supporting the usage of IMT, tapering corticosteroid dosage, and leading to new diagnoses of underlying systemic diseases [19]. The clinical approach described is different from our combined clinic. Practically, it may be difficult to have frequent multidisciplinary meetings for uveitis, but a combined clinic may be more feasible. To the best of our knowledge, this is the first analysis of clinical outcome data from a combined ophthalmology-rheumatology clinic in North America.

While corticosteroids remain the mainstay of treatment to control active uveitis, serious systemic and ocular side effects, such as hyperglycemia and glaucoma, respectively, render this treatment option practical for only a short period of time [20–22]. The need for long-term therapy for chronic uveitis and possible associated systemic autoimmune diseases, therefore, necessitates the prescription of immunomodulatory agents which requires co-management with rheumatologists for careful monitoring of disease activities and medication side effects [18, 19, 23]. Previous studies have demonstrated that one of the most important reasons for ophthalmologists to consult rheumatology is the need for therapeutic advice regarding systemic IMT [19]. A recent report concerns that the majority of ophthalmologists are not familiar with the usage of IMT in managing uveitis, leading to delays in corticosteroid tapering [24]. Our study showed that the combined uveitis clinic resulted in more frequent prescriptions of IMT and also a greater variety of immunomodulatory agents used to control uveitis and/or associated systemic autoimmune diseases. These joint efforts increased the number of patients at target dosage of IMT, demonstrating the effectiveness of the collaboration.

Although the change in visual acuity after the initial combined uveitis clinic was statistically significant, the improvement may not be clinically significant. Visual acuity may not be a meaningful marker for the benefits of the combined clinic as they can be affected by other ocular comorbidities. Interestingly, our data shows that the improvement in most of the other quantifiable markers of inflammation did not reach statistical significance. This is contrary to the expectation that offering a combined clinic would decrease the appointment burden and improve patient attendance, consequently allowing better inflammation control. However, from our experience, patients with uveitis tend to seek ophthalmological evaluation during an acute uveitis flare, i.e. when inflammation worsens, which can be controlled with local and systemic steroids. Therefore, inflammation control at predefined time points, as in our study (e.g. prior to and after the combined clinic) may not differ significantly. In our population, rheumatology appointments were more commonly missed, especially when the eye was asymptomatic, and the suboptimal dose of IMT in these patients would be a barrier to achieving long-term control. It is possible that there were more uveitis flares prior to implementing the combined clinic, but this was not easily quantifiable for our study duration.

There are several limitations in this study. Firstly, our sample size is relatively small. The small sample size, in combination with the large variation of patients who had uveitis, increases the chance of type 2 error in our study. This may partially account for the lack of significant differences in ocular inflammatory variables after the initial combined clinic visit. Secondly, the short duration of follow-up time in our study limits the potential for revealing the benefits of the combined clinic. It is possible that as follow-up duration increases, a greater proportion of patients will achieve the target dose of IMT, and the reduction of average systemic corticosteroid dose will reach statistically and clinically significance. Thirdly, the retrospective nature of the study was not able to control confounders and limits the definiteness of the conclusion. However, given the likelihood of improved IMT management found in this study, it may not be appropriate to randomize patients to combined versus separate ophthalmology and rheumatology clinics. Rather, it may be preferable to compare outcomes in institutions where both combined and separate uveitis-rheumatology clinics exist. Finally, 85.1% of our patients are African American. Although the exact demographic and distribution of systemic associations may not be the same in other cohorts, we believe the findings of improved IMT management in a combined clinic is likely generalizable.

## Conclusion

The primary goals of uveitis management are to better control disease activity and prevent vision deterioration while frequently transitioning patients from corticosteroid therapy to IMT. This study is the first to show benefits in improvement in IMT management in a combined ophthalmology-rheumatology clinic compared to when patients were separately managed. If feasible, this would be the preferred approach to manage uveitis in practices with high volume of uveitis patients.

## Data Availability

The datasets used and/or analyzed during the current study are available from the corresponding author on reasonable request.

## Abbreviations

BCVA: Best corrected visual acuity
IMT: Immunomodulatory therapy
MUST: The multicenter uveitis steroid treatment trial
FOCUS: The fundamentals of care for uveitis
AC: Anterior chamber
SUN: Standardization of Uveitis Nomenclature
